# Cross-Cultural Adaptation and Clinical Validation of TIME Criteria to Detect Potentially Inappropriate Medication Use in Older Adults: Methodological Report from the TIME International Study Group

**DOI:** 10.1007/s40266-024-01164-3

**Published:** 2024-12-17

**Authors:** Gulistan Bahat, Tugba Erdogan, Busra Can, Serdar Ozkok, Birkan Ilhan, Asli Tufan, Mehmet Akif Karan, Athanase Benetos, Antonio Cherubini, Michael Drey, Doron Garfinkel, Jerzy Gąsowski, Anna Renom-Guiteras, Marina Kotsani, Lisa McCarthy, Graziano Onder, Farhad Pazan, Karolina Piotrowicz, Paula Rochon, Georg Ruppe, Wade Thompson, Eva Topinkova, Nathalie van der Velde, Mirko Petrovic

**Affiliations:** 1 Division of Geriatrics, Department of Internal Medicine, Istanbul Medical Faculty, Istanbul University, Çapa, 34093 Istanbul, Turkey; 2 Division of Geriatrics, Department of Internal Medicine, Marmara University Medical School, Pendik, Istanbul, Turkey; 3 Division of Geriatrics, Department of Internal Medicine, Liv Hospital, Istanbul, Turkey; 4 Pôle « Maladies du Vieillissement, Gérontologie et Soins Palliatifs », and INSERM DCAC u1116, Université de Lorraine, CHRU-Nancy, 54000 Nancy, France; 5 Geriatria, Accettazione Geriatrica e Centro di Ricerca Per l’invecchiamento, IRCCS INRCA, Ancona, Italy; 6 Department of Medicine IV, LMU University Hospital, LMU Munich, Munich, Germany; 7 Center for Appropriate Medication Use, Sheba Medical Center, Tel Hashomer, Ramat Gan, Israel; 8 Department of Internal Medicine and Gerontology, Jagiellonian University Medical College, University Hospital, 2 Jakubowskiego St., Building I, 5th Floor, 30-688 Kraków, Poland; 9 Department of Geriatric Medicine, Hospital del Mar, Barcelona, Spain; 10 Pôle « Maladies du Vieillissement, Gérontologie et Soins Palliatifs », Université de Lorraine, CHRU-Nancy, Nancy, France; 11 Leslie Dan Faculty of Pharmacy, University of Toronto, Toronto, Canada; 12 Fondazione Policlinico Gemelli IRCCS and Università Cattolica del Sacro Cuore, Rome, Italy; 13 Ehemals Institut für Klinische Pharmakologie, Medizinische Fakultät Mannheim, Universität Heidelberg, Theodor-Kutzer-Ufer 1-3, 68167 Mannheim, Germany; 14 Women’s Age Lab and Women’s College Research Institute, Women’s College Hospital, Toronto, ON Canada; 15 European Geriatric Medicine Society (EUGMS), Vienna, Austria; 16 Department of Anesthesiology, Pharmacology, and Therapeutics, Faculty of Medicine, University of British Columbia, Vancouver, BC Canada; 17 Department of Geriatrics and Internal Medicine, First Faculty of Medicine, Charles University, General Faculty Hospital, Prague, Czech Republic; 18 Faculty of Health and Social Sciences, South Bohemian University, Ceske Budejovice, Czech Republic; 19 Internal Medicine, Section of Geriatric Medicine, Amsterdam UMC Location University of Amsterdam, Amsterdam, The Netherlands; 20 Amsterdam Public Health, Aging and Later Life, Amsterdam, The Netherlands; 21 Department of Internal Medicine and Paediatrics, Faculty of Medicine and Health Sciences, University of Ghent, Ghent, Belgium; 22 Department of Clinical and Molecular Sciences, Universita Politecnica delle Marche, Ancona, Italy

## Abstract

**Background:**

Various explicit screening tools, developed mostly in central Europe and the USA, assist clinicians in optimizing medication use for older adults. The Turkish Inappropriate Medication use in oldEr adults (TIME) criteria set, primarily based on the STOPP/START criteria set, is a current explicit tool originally developed for Eastern Europe and subsequently validated for broader use in Central European settings. Reviewed every three months to align with the latest scientific literature, it is one of the most up-to-date tools available. The tool is accessible via a free mobile app and website platforms, ensuring convenience for clinicians and timely integration of updates as needed. Healthcare providers often prefer to use their native language in medical practice, highlighting the need for prescribing tools to be translated and adapted into multiple languages to promote optimal medication practices.

**Objective:**

To describe the protocol for cross-cultural and language validation of the TIME criteria in various commonly used languages and to outline its protocol for clinical validation across different healthcare settings.

**Methods:**

The TIME International Study Group comprised 24 geriatric pharmacotherapy experts from 12 countries. In selecting the framework for the study, we reviewed the steps and outcomes from previous research on cross-cultural adaptations and clinical validations of explicit tools. Assessment tools were selected based on both their validity in accurately addressing the relevant issues and their feasibility for practical implementation. The drafted methodology paper was circulated among the study group members for feedback and revisions leading to a final consensus.

**Results:**

The research methodology consists of two phases. Cross-cultural adaptation/language validation phase follows the 8-step approach recommended by World Health Organization. This phase allows regions or countries to make modifications to existing criteria or introduce new adjustments based on local prescribing practices and available medications, as long as these adjustments are supported by current scientific evidence. The second phase involves the clinical validation, where participants will be randomized into two groups. The control group will receive standard care, while the intervention group will have their treatment evaluated by clinicians who will review the TIME criteria and consider its recommendations. A variety of patient outcomes (i.e., number of hospital admissions, quality of life, number of regular medications [including over the counter medications], geriatric syndromes and mortality) in different healthcare settings will be investigated.

**Conclusion:**

The outputs of this methodological report are expected to promote broader adoption of the TIME criteria. Studies building on this work are anticipated to enhance the identification and management of inappropriate medication use and contribute to improved patient outcomes.

**Supplementary Information:**

The online version contains supplementary material available at 10.1007/s40266-024-01164-3.

## Key Points

**Table:** 

Turkish Inappropriate Medication use in oldEr adults (TIME) criteria is an explicit screening tool to assist clinicians in the management of inappropriate medication use. The TIME criteria tool is reviewed and updated every three months to incorporate the latest evidence from the literature, ensuring it remains current and relevant for clinical practice. Its accessibility is enhanced through freely available mobile and web applications.
An up-to-date and easily accessible tool is urgently needed in many countries that rely on their mother tongues in clinical practice.
This paper describes the protocol for cross-cultural adaptation, language and clinical validation of TIME criteria for broader recognition and improved management of inappropriate medication use across different countries.

## Background and Rationale

Due to a high number of chronic diseases and geriatric syndromes, older adults receive many medications, which increases their risk for polypharmacy and inappropriate medication use, both of which are well-known risk factors for adverse drug reactions (ADRs). Further, frailty, a clinical syndrome that becomes more common with advancing age, causes heightened vulnerability to medications by impairing drug metabolism [1, 2]. Adverse drug reactions are a rising cause of mortality, morbidity and disability in older adults and are considered a significant healthcare issue [3–5].

Inappropriate medication use (IMU) refers to the use of definitively inappropriate medications, considering the specific clinical condition of a patient. In contrast, potentially inappropriate medication (PIM) use refers to medications that are generally inappropriate for most older individuals but may be appropriate in certain cases after a thorough clinical evaluation by the attending clinician. Both explicit (criteria-based) screening tools and implicit (patient-based) evaluation methods have been developed to help clinicians identify and manage medications for older adults. Explicit tools offer standardized guidelines for PIM detection based on general criteria, making them easier to apply and less time consuming. Implicit tools, on the other hand, involve comprehensive, patient-specific evaluations that require a clinician’s judgment. As a result, most studies on IMU rely on explicit tools that classify medications as PIM rather than definitively inappropriate IMU.

The negative medical, economic, and social consequences associated with PIM use render medication optimization an essential component of geriatric care [6–9]. Research to date demonstrates an association between optimizing medications and improved clinical outcomes [10–13]. However, PIM is still commonly encountered, especially among older adults who are institutionalized and have comorbidities [14–18]. Of note, older adults are also at risk of being denied clinically indicated medications without a justified reason. Therefore, potentially inappropriate prescribing (PIP) encompasses not only over-prescribing and mis-prescribing but also under-prescribing [19]. Clinicians involved in the medication management of older adults should also consider this latter aspect of inappropriate medication use (i.e., underuse) to ensure comprehensive and balanced care in their patients.

The most commonly used explicit criteria sets are the American Geriatrics Society Beers Criteria [20, 21] and the Screening Tool of Older Persons’ potentially inappropriate Prescriptions/Screening Tool to Alert to Right Treatment (STOPP/START) tools [22, 23], both of which have been recently revised [21, 23]. A tool specific to deprescribing fall-risk increasing drugs (FRIDs) [24, 25], STOPPFall (Screening Tool of Older Persons Prescriptions in older adults with high fall risk) has been developed by an expert group from Europe [26]. ThinkCascades is a tool to help recognize the most important prescribing cascades in clinical practice [27]. Deprescribing tools specific to frail older adults and adults with limited life expectancy are also available [28–30].

Prescribing habits and the availability of medications vary significantly across countries. In addition, the explicit screening tools need to be updated regularly to reflect emerging scientific evidence. To meet the needs of Eastern European region and also address the global need for an up-to-date explicit screening tool, the Turkish Inappropriate Medication use in oldEr adults (TIME) criteria was introduced in Turkey in 2020 [31]. During its development, the early draft was primarily based on the STOPP/START criteria set (due to its European origin, recognition of the dual nature of PIMs and practical statement system) [32], with additional input from the CRIME criteria (given their focus on the needs of complex older adults) [33]. The subtitles STOPP and START were designed to maintain harmony with the STOPP/START criteria set, as it shaped the core structure and provided a clear classification system facilitating easier implementation in practice. To enhance its reliability, applicability and broader acceptance beyond geriatricians, TIME incorporated input from a wider range of specialists involved in management of older adults, unlike most existing tools developed solely by geriatricians and pharmacologists. The process involved the participation of 27 academicians from various medical specialties (TIME National Working Group-2020, see the supplementary information) with comprehensive review of the PubMed, EMBASE, and Cochrane Library databases. Based on the recent findings in the literature and common problematic prescribing practices/available medications in the region, 43 new criteria were introduced, 17 were removed, and a total of 60 criteria were adapted or modified from the initial draft by the TIME study group. The final version consisted of 153 criteria, including 112 TIME-to-STOP and 41 TIME-to-START criteria [31]. The TIME criteria subsequently underwent further validation for international use through a Delphi study in 2021, expanding its applicability beyond Eastern Europe [34]. The validated TIME list comprised 134 criteria (101 TIME-to-STOP and 33 TIME-to-START criteria) [34]. The TIME-to-STOP criteria identify medications and supplements that may pose potential side effects for older adults, while the TIME-to-START criteria highlight medications and nutritional supplements that are beneficial but overlooked in clinical practice, often due to the patient's advanced age without a valid reason. Both the use of medications listed in the TIME-to-STOP criteria and the omission of those recommended in the TIME-to-START criteria are explicitly defined as PIP.

The TIME criteria can be accessed and downloaded from its official website: https://www.timecriteria.com. Users can search the criteria by organ system, disease, or specific medications. The TIME criteria are reviewed every three months by the extended TIME National Working Group (see the supplementary information) to incorporate up-to-date recommendations. This process involves assessing the need for modifications, additions, or removals based on the latest scientific evidence. Any changes to the criteria are backed by appropriate references, while unchanged criteria continue to reflect current best practices in prescribing and deprescribing. In addition to its web-based accessibility, the TIME criteria are also available as a free mobile application for health professionals (https://apps.apple.com/tr/app/time-criteria/id1560761256 and https://play.google.com/store/search?q=TIME%20criteria&c=app). This feature, offered by only a few other explicit tools, ensures convenience for clinicians and facilitates the timely integration of updates. Potentially inappropriate medication use as determined by the TIME criteria has been shown to have a potential association with mortality, highlighting its possible impact on patient outcomes [35]. Nevertheless, further studies in other countries are warranted to assess the effectiveness of TIME in detecting PIM use and its benefit in reducing adverse outcomes in clinical practice.

There are many different languages in Europe and around the world. While English is the most commonly used language in medical research, many clinicians do not have an advanced proficiency in English. Moreover, many specifically prefer reading medical recommendations in their native languages, as it allows them to better understand the content and feel more confident when considering their implementation in clinical practice. As such, language barriers can hinder the integration of well-documented recommendations into clinical care, highlighting the need for tools in native languages to optimize medication management. There are tools specific to Germany (PRISCUS [36] and FORTA [37]), Belgium (GheOP3S [38]), Norway (NORGEP [39]) and France [40]. However, to the best of our knowledge, most explicit tools are only available in either the official language of the country they originate from and/or in English. Although some efforts have been made to address this, such as direct translations of the original tools (some by excluding unavailable drugs in that market), (e.g., for the STOPP/START criteria [41, 42]), these translations often lack the nuance required to accommodate the diverse healthcare practices and contexts of different regions effectively. In addition to overcoming language barriers, we considered it important to take key factors such as locally available drugs, PIP profile, and regional prescribing habits into account to better address the specific needs of different regions—an approach that was applied during the development of the TIME criteria. To address these challenges, we decided to launch a series of studies to perform cross-cultural and language validation of TIME criteria in a variety of frequently used languages allowing regions or countries to make modifications to existing criteria or introduce new ones as long as these adjustments are supported by current scientific evidence. Given the universal nature of the criteria, such changes are expected to be minimal and likely driven by local prescribing habits and the availability of specific medications. As such, the initiative aims to create country-specific adaptations rather than simple translated versions. We also aimed to launch clinical validation studies in different settings to evaluate the potential of TIME criteria in detecting PIM use and reducing adverse outcomes in clinical practice.

Recognized experts in geriatric pharmacotherapy, including those who participated in the Delphi validation, were invited to join the International Study Group for cross-cultural adaptation and clinical validation studies. Twenty-four experts from 12 different countries accepted the invitation, forming the TIME International Study Group (see the supplementary information). As a component and first element of this initiative, we were required to develop a standardized methodological report to ensure consistency and comparability, harmonizing these studies across different regions and settings, while allowing for adjustments to accommodate local needs and practices. To fulfill this objective, we reviewed the steps and the outcomes of previous cross-cultural adaptation and clinical validation studies. Validity and feasibility were taken into consideration when determining the assessment tools. The drafted methodology was then shared with the members of the International Study Group via email and revised according to their feedback and recommendations. The present article presents the final protocol to be used as a standard model by the study teams.

## Validation Process

### Cross-Cultural Adaptation and Language Validation

**Fig. 1:**
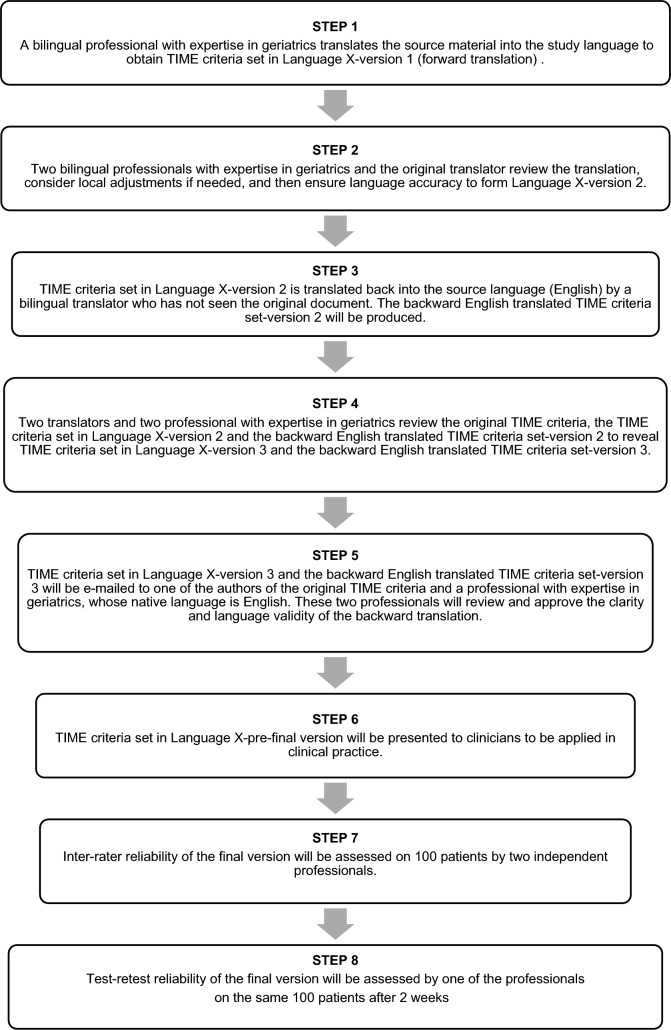
Flowchart outlining the translation, cross-cultural adaptation and reliability testing of TIME criteria. *TIME* Turkish Inappropriate Medication use in oldEr adults

Cross-cultural adaptation and language validation should be conducted to ensure the translated versions of TIME criteria convey the intended meaning. The methodology of validation is based on WHO recommendations for scientific validation [43]. The process involves three bilingual geriatricians or professionals with expertise in geriatrics, with the study language as their first language spoken and one bilingual backward translator (Fig. 1). Physicians, pharmacists, nurse practitioners with expertise in geriatrics are eligible for conducting the validation. The original source material is the TIME criteria in English. Since the TIME criteria set is reviewed every three months, the version used for cross-cultural and language adaptation should be the latest one available at the time, accessible at https://www.timecriteria.com.

*Step 1* First, a bilingual professional with expertise in geriatrics translates the source material (forward translation) into the study language to obtain TIME criteria set in Language X-version 1.

*Step 2* The second step involves two bilingual professionals with expertise in geriatrics (at least one with prior experience in a validation study) reviewing the proposed translation. Together with the original translator, these professionals form the study team for Language X and can incorporate adjustments to the existing criteria or introduce new ones based on local prescribing practices and available medications, as long as these changes are supported by current scientific evidence and relevant references. The group will then work to ensure the accuracy of the translation, forming the Language X-version 2 of the TIME criteria set. It should be noted that conceptual equivalence is more important than word-to-word translation.

*Step 3* The TIME criteria set in Language X-version 2 is translated back into the source language (English) by a bilingual translator who has not seen the original document. This bilingual translator should either have English as their native language or should be a lecturer in an English Language Department. The backward English translated TIME criteria set-version 2 will thus be produced.

*Step 4* Two translators and two professionals with expertise in geriatrics review the original TIME criteria, the TIME criteria set in Language X-version 2 and the backward English translated TIME criteria set-version 2 to reveal TIME criteria set in Language X-version 3 and the backward English translated TIME criteria set-version 3.

*Step 5* The TIME criteria set in Language X-version 3 and the backward English translated TIME criteria set-version 3 will be e-mailed to one of the authors of the original TIME criteria and a professional with expertise in geriatrics, whose native language is English. The two experts will review and approve the clarity and language validity of the backward translation.

*Step 6* The TIME criteria set in Language X-pre-final version will be presented to clinicians to be applied in clinical practice. If the clinicians have no difficulty comprehending and using the translated TIME criteria, TIME criteria set in Language X-final version will be complete.

*Step 7* Inter-rater reliability of the final version will be assessed on 100 patients by two independent professionals with expertise in geriatrics [44]. Assessment of medical records may be preferable to in-person visits.

*Step 8* The test-retest reliability of the final version will be assessed by one of the experts on the same 100 patients after 2 weeks [44]. Assessment of medical records may be preferable to in-person visits.

### Clinical Validation

**Fig. 2:**
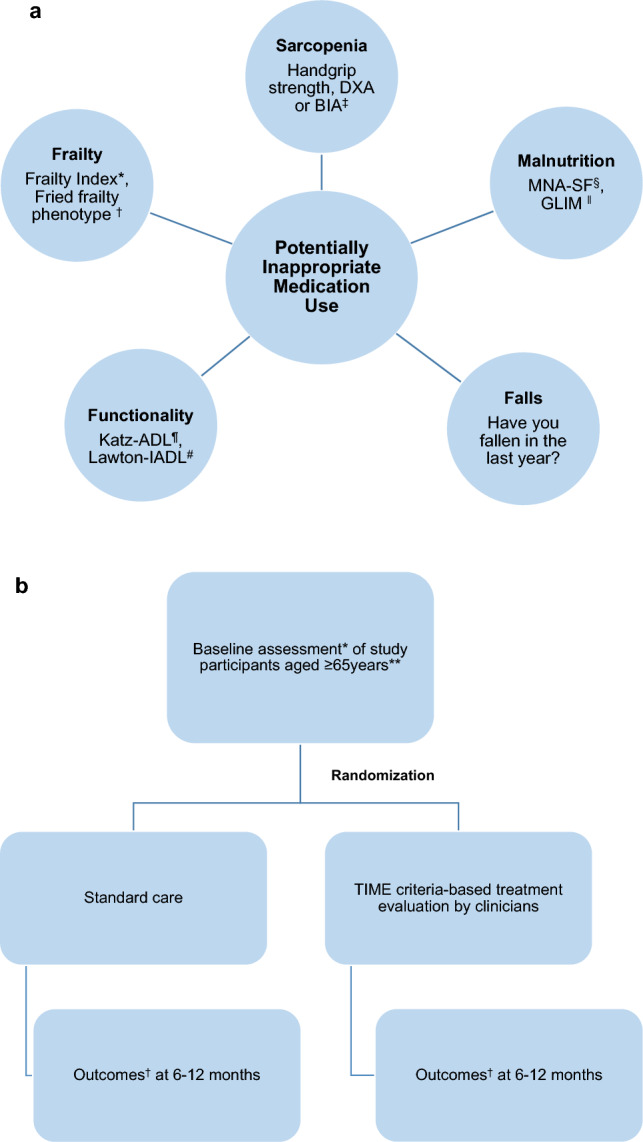
a Examples of the recommended tools to evaluate geriatric syndromes in relation to potentially inappropriate medication use identified by the TIME criteria. *Frailty Index [53]. ^†^ Fried frailty phenotype [52]. ^‡^ *BIA* bio-impedance analyzer, *DXA* dual-energy X-ray absorptiometry. ^§^ Mini-Nutritional Assessment-Short Form (MNA-SF) [50]. ^‖^ The Global Leadership Initiative on Malnutrition (GLIM) criteria [51]. ^¶^ Katz Activities of Daily Living Scale (ADL) [48]. ^#^ Lawton Instrumental Activities of Daily Living Scale (IADL) [49]. Depending on their clinical experience, the center conducting the research may prefer alternative assessment methods over those proposed in this paper. Examples of other tools in the literature are the Clinical Frailty Scale (CFS) [54] and Simpler Modified Fried Frailty Scale [55] for the assessment of frailty, Barthel Index [66] for the activities of daily living, and Nutritional Risk Screening 2002 (NRS-2002) [67], Malnutrition Universal Screening Tool (MUST) [68] and Subjective Global Assessment (SGA) [69] for the assessment of malnutrition. **b** Participant randomization and outcome assessment (standard care vs TIME criteria-based treatment evaluation by clinicians). *Baseline assessment will encompass various geriatric syndromes including cognition, functionality, nutritional status, frailty, sarcopenia, urinary incontinence, constipation, falls, orthostatic hypotension, chronic pain, dysphagia, vision and hearing problems, depression, and sleep disorders, although the specific syndromes assessed may vary by study. **The sample size will be calculated individually for each specific study, considering its design, outcomes of interest, and setting. ^†^Outcomes include hospital admission rates, quality of life, number of regular medications, the presence of geriatric syndromes, and mortality, depending on the study design. *TIME* Turkish Inappropriate Medication use in oldEr adults

In the clinical validation studies, our primary aims are to: (1) determine the prevalence of PIM using TIME criteria during the assessment of older patients in different healthcare settings, (2) examine whether the clinical integration of TIME criteria would improve patient outcomes as compared to the previous visit (e.g., 6–12 months previously), (3) examine whether the clinical integration of TIME criteria (intervention group) would improve outcomes when compared to standard care (control group) prospectively. In the clinical validation studies, our secondary aims are to: (1) determine whether PIM use per TIME criteria constitute(s) the reason for current hospital admission, (2) determine the cross-sectional association between PIM detected by TIME criteria and geriatric syndromes (Fig. 2a), (3) examine whether integration of TIME criteria would improve prescribing patterns at 6–12 months of follow-up, i.e., adherence to the application of TIME criteria. The outcomes include number of hospital admissions, quality of life, number of regular medications (including over-the-counter medications), geriatric syndromes and mortality at 6–12 months of follow-up (only for participants who will be followed prospectively). The geriatric syndromes of interest include cognition, functionality, nutritional status, frailty, sarcopenia, urinary incontinence, constipation, falls, orthostatic hypotension, chronic pain, dysphagia, vision and hearing problems, depression, and sleep disorders. The patients will be randomized into two groups upon presentation using a web-based application designed to perform random sampling (www.random.org) [45]. The first group will receive standard care, while the intervention group will have their treatment evaluated by clinicians who will review the TIME criteria and consider its recommendations (Fig. 2b).

#### Patient Population

*Inclusion criteria* The participants should be older adults aged ≥ 65 years in a variety of healthcare settings (inpatient, outpatient, long-term care, home care, etc.). A minimum number of 100 participants are required for the cross-cultural adaptation and language validation phase (corresponding to inclusion minimum 2 participants for each TIME criterion) [44]. As there will be various studies in different settings and evaluating diverse outcomes, the sample size will be individually calculated for each specific study, factoring in its design, outcomes of interest, and setting.

*Exclusion criteria* Patients with severe dementia (Mini-Mental State Examination score < 10), patients with limited life expectancy (< 6 months), patients actively participating in another clinical trial of a medicinal product, and non-accidental overdose/self-harm.

#### Parameters and Assessment Tools

Date of birth, sex, educational status, height (cm) and weight (kg) of each study participant will be recorded. The presenting complaint will be documented for out-patients, while hospital admission diagnosis will be documented for in-patients. Laboratory tests that are part of the patients’ overall care plan will be recorded if performed within two days of initial evaluation. The following tests will be recorded if available: C-reactive protein, hemoglobin, creatinine, albumin, fasting blood glucose, vitamin D, calcium, thyroid-stimulating hormone and lipids. The values will be interpreted according to the reference ranges of the study center’s laboratory.

Cognitive screening will be performed by the Mini-Mental State Examination (MMSE), which is a test consisting of 11 items grouped under 5 main areas; orientation, recording memory, attention and calculation, recall and language. The total score is 30 points and the cut-off point indicating cognitive impairment is 24 points [46]. The Montreal Cognitive Assessment (MoCA) [47], may also be preferred, depending on the clinical experience of the study center.

Functional status will be assessed by the 6-item Katz Activities of Daily Living Scale-ADL (min 0, max 6 points) [48] and 8-item Lawton Instrumental Activities of Daily Living Scale-IADL (min 0, max 8 points) [49]. The score for each item is 0 if the patient is dependent and 1 if the patient is independent for the activity. Higher total scores indicate better independence.

Nutritional status will be screened with the Mini-Nutritional Assessment-Short Form (MNA-SF) [50]. MNA-SF score ranges between 0 and 14. A score of 0–7 is considered "malnutrition", a score of 8–11 is considered "at risk of malnutrition", and a score of 12 and above is considered "normal". The Global Leadership Initiative on Malnutrition (GLIM) criteria may be used to diagnose malnutrition [51]. The GLIM criteria consists of three phenotypic and two etiological criteria. Phenotypic criteria are weight loss, low body mass index (BMI), and low muscle mass. Etiological criteria are reduced food intake/assimilation and disease burden/inflammation. To diagnose malnutrition at least one phenotypic criterion and one etiologic criterion should be present. The GLIM criteria also provide staging of malnutrition as moderate (stage 1) versus severe (stage 2) using the phenotypic criteria. The components of *Stage 1 (moderate)* malnutrition are presence of 5–10% weight loss in the past 6 months or 10–20% weight loss beyond 6 months or low BMI (< 20 kg/m^2^ if aged < 70 years, <22 kg/m^2^ if aged ≥ 70 years) or mild to moderate deficit in muscle mass. Components of *Stage 2 (severe)* malnutrition are >10% weight loss in the past 6 months or >20% weight loss beyond 6 months or low BMI (< 18.5 kg/m^2^ if aged < 70 years, <20 kg/m^2^ if aged ≥ 70 years) or severe deficit in muscle mass [51].

Frailty will be evaluated either with the Fried frailty phenotype (FP) [52] or the deficit-accumulation frailty index (FI) [53]. The FP questions unintentional weight loss, exhaustion, low grip strength, slow walking speed, and low physical activity, where three or more positive answers indicate frailty [52]. The FI calculates the proportion of accumulated health deficits in an individual [53]. However, some centers may find alternative tools easier to implement in their routine clinical practice. In this case, the Clinical Frailty Scale (CSF) [54] may be used instead of the FI, or the Simpler Modified Fried Frailty scale [55] may replace the FP. The CFS has a judgment-based scoring between 1 (very fit) and 9 (terminally ill) that facilitates utilization [54]. The Simpler Modified Fried Frailty scale consists of five questions on weight loss, weakness, exhaustion, low physical activity level, and slow walking speed. Scores range from 0 to 5, where 3–5 represents frailty and 1–2 represents pre-frailty [55]. Depending on the experience of the study center, other reliable assessment tools in the literature including Gérontopôle Frailty Screening Tool (GFST) [56] and the FRAIL scale [57] may also be preferred.

Sarcopenia will be screened by SARC-F [58]. If resources allow, a more comprehensive assessment for sarcopenia will be conducted. Although the authors acknowledge the Global Leadership Initiative on Sarcopenia (GLIS) definition as the expected operational standard, the study will utilize the revised consensus of European Working Group on Sarcopenia in Older People (EWGSOP2) [59] operational definition until the GLIS criteria are fully documented. Probable sarcopenia will be assessed with handgrip strength (HGS) measurement [59].

The HGS will be measured with a hydraulic hand dynamometer in a sitting position, with the elbow in 90° flexion, wrist in the neutral position. Participants will be asked to apply the maximum grip strength with both left and right hands, three times for both sides. The maximum grip strength performed will be accepted as the HGS value. The cut-offs for HGS will be considered as 27/16 kg in males and females, respectively.

Depending on the study center, muscle mass evaluations will be made either with a bio-impedance analyzer or dual-energy X-ray absorptiometry (DXA). The EWGSOP2 suggested cut-offs will be used in the analysis. Usual gait speed will be calculated by the 4-meter walking test, with speed measured either with a stopwatch or with an electronic device and the ≤ 0.8 m/s as the cut-off for severe sarcopenia.

Geriatric syndromes including urinary incontinence, constipation, falls, orthostatic hypotension, chronic pain, dysphagia, vision and hearing problems, depression and sleep disorders will also be evaluated.

Urinary incontinence will be defined as the complaint of any involuntary leakage of urine in the past 12 months (A yes/no question regardless of the type of incontinence). Presence of constipation will be questioned as follows: ‘Do you have unsatisfactory defecation due to infrequent stools, difficult stool passage, or both? Do you have symptoms of straining, difficulty expelling stool, a sense of incomplete evacuation, hard or lumpy stools, prolonged time to pass stool, or a need for manual maneuvers to pass stool, which has been present for at least 3 of the prior 12 months?’ “Have you ever fallen in the last year?” will be asked to all participants for fall risk assessment. Orthostatic hypotension (OH) will be defined as a 20 mm Hg or greater decline in systolic blood pressure (BP) and/or 10 mm Hg or greater decline in diastolic BP when changing from a supine or sitting to standing position, measured at 1 and 3 minutes upon standing. For chronic pain assessment, participants will be asked whether they have been experiencing musculoskeletal pain ongoing for ≥ 3 months. The severity of pain will be measured by the Visual Analogue Scale (VAS). The Eating Assessment Tool (EAT-10), which asks about oropharyngeal, esophageal, liquid and solid dysphagia with 10 simple questions, will be used for dysphagia screening [60]. For each question, 0 is considered normal, while 4 points indicate severe problem. Accordingly, higher scores are suggestive of a swallowing disorder. Two different thresholds have been identified for the EAT-10, where a score ≥ 3 denotes positive screening, and a score > 15 denotes significant risk of aspiration. A claims-based vision and hearing assessment will be used for practicality. For depression screening, the 5-item Geriatric Depression Scale (GDS-5) will be administered [61]. Sleep disorders will be evaluated by the Insomnia Severity Index (ISI) [62]. This questionnaire categorizes total scores into 0–7 = no significant sleep disorder, 8–14 = subthreshold insomnia, 15–21 = moderate insomnia, and 22–28 = severe insomnia.

Quality of life will be assessed by the EuroQoL five-dimensional questionnaire (EQ-5D) [63]. The five domains of EQ-5D include mobility, self-care, usual activities, pain/discomfort and anxiety/depression.

Although the above-mentioned tests have not been validated for use over the telephone, follow-up may be planned as telephone interviews when face-to-face assessment is not possible.

Depending on their clinical experience, the center conducting the research may prefer alternative assessment methods over those proposed in this paper (Fig. 2a).

### Statistical Analysis

The variables will be investigated using visual (histograms and probability plots) and analytical methods to determine whether they are normally distributed. Numerical variables will be reported as mean ± standard deviation for normally distributed variables and as median (minimum-maximum) for skew-distributed continuous variables. Categorical variables will be shown as frequencies. For the cross-cultural and language validation of TIME criteria, reliability will be assessed by internal consistency, inter-rater and test–retest analyses. The level of agreement assessed by kappa coefficient will be defined as follows: kappa coefficient > 0.90: almost perfect agreement, between 0.80 and 0.90: strong agreement, 0.60–0.79: moderate agreement, 0.40–0.59: weak agreement, 0.21–0.39: minimal agreement, and 0.00–0.20: no agreement [64]. Additionally, considering the total number of TIME criteria as a numerical variable, the inter-rater reliability and test-rest reliability can be tested by intra-class correlation coefficient (ICC) provided that the sum of START and STOP criteria are normally distributed. The ICC estimates and their 95% confidence intervals (CIs) will be calculated based on a single measurement, absolute agreement, 2-way mixed-effects model. Reliability assessed by ICC estimates will be defined as follows: ICC estimate > 0.90: excellent reliability, between 0.75 and 0.9: good reliability, 0.5–0.75: moderate reliability, < 0.5: poor reliability [65]. Finally, internal consistency will be tested by Cronbach’s alpha coefficient, where a value greater than 0.70 indicates a high level of internal consistency.

Two groups will be compared with independent sample t-test or Mann Whitney *U* test when necessary. Chi-square test with Yates correction and Fisher’s exact test will be used for 2 × 2 contingency tables when appropriate for non-numerical data. Correlations between numerical parameters will be analyzed with Pearson’s or Spearman’s rho correlation test when necessary. Groups were compared with analysis of variance (ANOVA) as necessary. Comparisons involving more than two groups will be made by Kruskal Wallis-H analysis of variance when the distribution is abnormal. Tukey Honestly Significant Difference (HSD) test will be used for post hoc comparisons. The variables detected as significant in univariate analyses will be analyzed with appropriate regression models depending on the nature of the dependent variable and study design. For binary outcomes (e.g., presence or absence of a condition), logistic regression analysis will be performed to estimate odds ratios (ORs) and 95% CIs. For continuous outcomes, linear regression will be used to determine associations between predictor variables and the dependent variable, with results presented as regression coefficients (β) and corresponding 95% CIs. For time-to-event outcomes, Cox proportional hazards regression analysis will be conducted to estimate hazard ratios (HRs) and 95% CIs. All models will be adjusted for potential confounders, and multicollinearity will be evaluated using correlation analyses (Pearson, Spearman, or Kendall’s tau-b). Variables showing high multicollinearity will be excluded from the same regression models to ensure robustness. The *p* values less than 0.05 will be accepted as significant.

## Conclusion

The TIME criteria set is a practical tool to review medications and address common prescribing errors, regularly reviewed and updated as needed, and made efficient through its accessible free mobile and web applications. The effectiveness of TIME criteria in detecting potentially inappropriate medications (PIM) and improving outcomes in older adults needs to be tested through large-scale studies across different healthcare settings to explore its clinical impact.

This methodological report outlines the protocol for cross-cultural adaptation and language validation of the TIME criteria, aiming to facilitate its application across various regions and languages while maintaining consistency with evidence-based guidelines. These adaptations will help ensure the criteria's relevance to local prescribing practices and medication availability, while the language validation will provide healthcare professionals with access to a tool that optimizes medication use in their native language. Additionally, a comprehensive framework for clinical validation studies is presented to evaluate the tool’s effectiveness in various healthcare settings and patient populations. The outcomes of these studies will be presented in a series of investigation papers, which are expected to promote broader adoption of the TIME criteria. Studies building on this work are anticipated to enhance the identification and management of inappropriate medication use and contribute to improved patient outcomes.

## Supplementary Information

Below is the link to the electronic supplementary material.

Supplementary file1 (DOCX 20 kb)
