# Supplementary material for: Cross-Cultural Adaptation and Clinical Validation of TIME Criteria to Detect Potentially Inappropriate Medication Use in Older Adults: Methodological Report from the TIME International Study Group
Source: Drugs Aging. 2024 Dec 17;42(1):57–67. doi: 10.1007/s40266-024-01164-3 (PMC13139231; doi:10.1007/s40266-024-01164-3)
Supplement: Supplementary file 1 — Supplementary file1 (DOCX 20 kb) [file 40266_2024_1164_MOESM1_ESM.docx]

**Supplementary Information**

**Members of the TIME National Working Group – 2020**: Gulistan Bahat, Birkan Ilhan, Tugba Erdogan, Meltem Halil, Sumru Savas, Zekeriya Ulger, Filiz Akyuz, Ahmet Kaya Bilge, Sibel Cakir, Kutay Demirkan, Mustafa Erelel, Kerim Guler, Hasmet Hanagasi, Belgin Izgi, Ates Kadioglu, Ayse Karan, Isin Baral Kulaksizoglu, Ali Mert, Savas Ozturk, Ilhan Satman, Mehmet Sukru Sever, Tufan Tukek, Yagiz Uresin, Onay Yalcin, Nilufer Yesilot, Meryem Merve Oren, Mehmet Akif Karan.

**TIME National Working Group – 2024:** Gulistan Bahat, Mehmet Akif Karan, Birkan Ilhan, Tugba Erdogan, Serdar Ozkok, Meltem Halil, Zekeriya Ulger, Sumru Savas, Ilker Tasci, Ilhan Satman, Gulsah Yenidunya, Ozlem Soyluk Selcukbiricik, Filiz Akyuz, Mehmet Sukru Sever, Savas Ozturk, Ahmet Kaya Bilge, Samim Emet, Mustafa Erelel, Zuleyha Bingol, Ali Mert, Atahan Cagatay, Hasmet Hanagasi, Basar Bilgic, Nilufer Yesilot, Serpil Bulut, Ceren Alis, Isin Baral Kulaksizoglu, Sibel Cakir, Nese Direk Tecirli, Funda Gungor, İnci Sema Tas, Ates Kadioglu, Murat Dursun, Belgin İzgi, Serife Bayraktar, Ayse Karan, Tugba Aydin, Kutay Demirkan, Betul Okuyan, Zeynep Gunes Ozunal, Yagiz Uresin, Hasan Raci Yananli, Tufan Tukek, Kerim Guler, Alpay Medetalibeyoglu, Naci Senkal, Meryem Merve Oren.

**TIME International Study Group – 2024:** Gulistan Bahat, Tugba Erdogan, Busra Can, Serdar Ozkok, Birkan Ilhan, Asli Tufan, Mehmet Akif Karan, Athanase Benetos, Antonio Cherubini, Michael Drey, Doron Garfinkel, Jerzy Gąsowski, Anna Renom-Guiteras, Marina Kotsani, Lisa McCarthy, Graziano Onder, Farhad Pazan, Karolina Piotrowicz, Paula Rochon, Georg Ruppe, Wade Thompson, Eva Topinkova, Nathalie van der Velde, Mirko Petrovic.
